# Mineral and geochemical variability of the phosphorite deposits in the Duwi Formation, Western Desert, Egypt: Insights into paleoenvironment and physicochemical conditions

**DOI:** 10.1038/s41598-026-46266-7

**Published:** 2026-04-30

**Authors:** Gehad M. Saleh, Mokhles K. Azer, Diaa A. Saadawi, Sameh H. Negm, El Saeed R. Lasheen

**Affiliations:** 1 Nuclear Materials Authority, El Maadi, Cairo, Egypt; 2 National Research Centre, Cairo, Egypt; 3 Geology Department, Faculty of Science, Al-Azhar University, Cairo, Egypt

**Keywords:** Abu Tartur, Duwi Formation, Phosphorite, Apatite chemistry, Bulk rock analysis, Environmental sciences, Solid Earth sciences

## Abstract

This study explores the phosphorite deposits from the Lower Member of the Duwi Formation in Egypt, which forms part of the Mediterranean phosphorite belt extending from Africa to Asia. Detailed petrographical, mineralogical, and geochemical investigations were carried out to shed light on the prevailing physicochemical conditions and diagenetic history of these deposits. They are classified into three east-west trending belts, with significant economic occurrences located in the central facies belt, particularly along the Nile Valley and Red Sea shoreline. Geochemical analyses indicated that these phosphorites are predominantly composed of CaO (avg. 35.83 wt%) and P_2_O_5_ (avg. 25.8 wt%) with high average of detrital oxides like SiO_2_ (9.08 wt%), and Al_2_O_3_ (1.74 wt%) relative to seawater- derived phosphorite. The PAAS-normalized REEs plot shows that the examined phosphorites are characterized by slight negative Ce and positive Eu anomalies, with relatively enrichment of MREEs over LREEs and HREEs. These patterns and anomalies are inconsistent with typical seawater-derived phosphorites and instead point to detrital heavy mineral input. Sea-level changes during the Campanian-Maastrichtian periods are identified as the likely mechanism that the redeposition of pre-existing authigenic phosphorites into the current deposits within an oxic and dysoxic basin setting, as evidenced by their distinct chemical features. From an economic perspective, the high ∑REE + Y content (avg. 969 ppm) in these phosphorites is significant, as it surpasses the concentrations typical of seawater-derived deposits. Their economic potential is further highlighted by strong enrichment in light REEs (avg. 646 ppm) with substantial concentrations of both critical REEs (e.g., Nd = 153.89 ppm and Y = 182 ppm) and non-critical REEs (e.g., La = 154.36 ppm).

**Supplementary Information:**

The online version contains supplementary material available at 10.1038/s41598-026-46266-7.

## Introduction

**Fig. 1:**
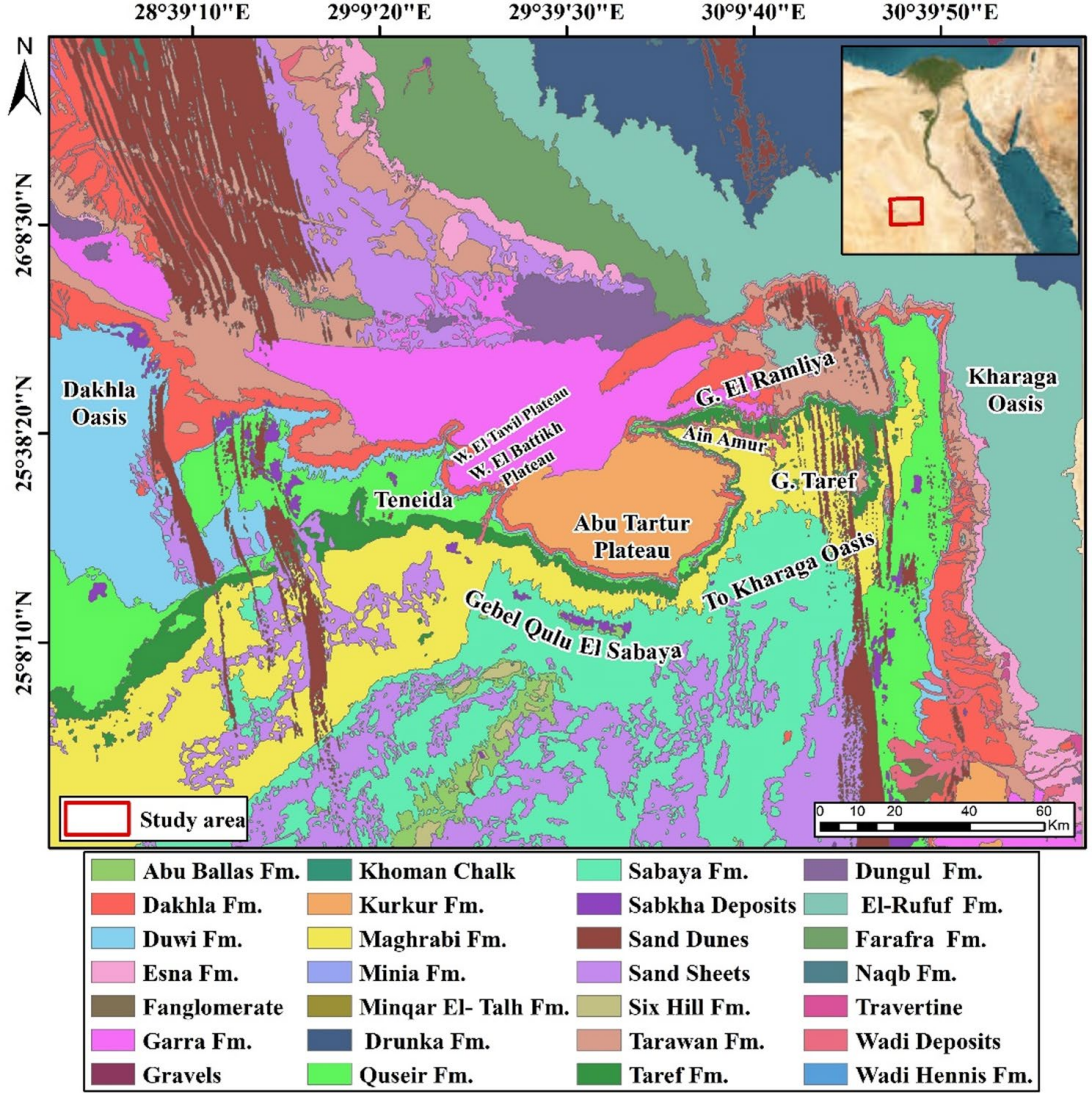
Abu Tartur map^17^ utilizing Arc GIS 10.4 and ENVI 5.3.

Phosphate rock is a phosphorus-rich (primarily expressed as P₂O₅) marine biochemical sedimentary rock. As a by-product of the extraction of trace element concentrations, it is utilized in high-tech businesses (such REEs) and in agriculture for fertilizer manufacture, making it one of the most significant raw materials^1–3^. Globally, phosphorite is found in both igneous and sedimentary rocks, but the most economically important deposits are of marine sedimentary origin. These marine deposits account for approximately 80% of global phosphate production, while igneous rocks contribute about 17%. The remaining small percentage comes from residual sedimentary and guano-type deposits^3^. The largest igneous source is the Devonian Khibina alkaline complex in Russia, with other major examples in Brazil and South Africa^1,4^. Sedimentary phosphorite deposits, which are more widespread, are found across the globe. Major economic phosphorite exist in the United States, Morocco, China, and Australia^1^. A particularly prolific belt of these sedimentary deposits extends across the Middle East and North Africa, from Morocco in the west to Iraq and Turkey in the east, encompassing countries like Algeria, Egypt, Jordan, and Saudi Arabia^5,6^. Sedimentary (phosphorite), igneous, and metamorphic rocks are widely exposed in Egyptian Desert^7–11^. The phosphorite deposits in Egypt belong to the Duwi Formation that represents part of the transcontinental Mediterranean phosphorite belt^12^, which links Arab countries in North Africa (west) with those in Western Asia (east). Egyptian phosphorite occurrences, classified by Hermina^13^ into three east-west-oriented belts, host economic resources exclusively within the central facies belt. This includes: Red Sea coastal sector (Safaga to Quseir), Nile Valley segment (Idfu to Qena), Western Desert province (Kharga-Dakhla oases; Abu Tartur area, Fig. 1)^13,14^. Due to their economic significance, the geochemistry of phosphorites, which is primarily composed of carbonate fluorapatite (francolite), has been extensively studied. Variability in francolite composition reflects differences in original deposition and diagenetic modification. Globally, sedimentary phosphorites consist of francolite, though fluorine content increases with age^15^. For example, Upper Cretaceous-Lower Tertiary francolite contains 3–5 wt% F (F/P₂O₅ ~ 0.11), contrasting sharply with modern human bone (fluoride levels in the tens of ppm).

Cathcart^16^ identified phosphorites as natural uranium sources, where U is enriched during deposition and diagenesis via seawater interaction. Uranium (30–300 ppm; up to 500 ppm in marine-reworked phosphorite) resides primarily in apatite, existing as U⁴⁺ and U⁶⁺. U⁴⁺ substitutes for Ca²⁺ in the apatite lattice due to similar ionic radii, while U⁶⁺ adsorbs onto mineral surfaces. Notably, Abu Tartur phosphorite hosts Egypt’s highest REE concentrations (up to 2,000 ppm)^17,18^, but its U content (24 ppm) is anomalously low compared to El Sibaiya (85 ppm) and Red Sea deposits (68 ppm).

Lasheen^17^ studied the geochemical, geological, and radiological risk of the Gebel Qulu El Sabaya phosphorites to assess their ability for variable applications. The radiological parameters reflect that the continuous exposure to these deposits could threaten human organ wellness. Likewise, Fathy^19^ studied the geological and radiological risk of the Hamadat phosphorites. They noticed that the Hamadat phosphorite have high radioactivity, which is chiefly attributable to the widespread presence of phosphatic components (apatite).

The present work aims to characterize the Abu Tartur phosphorite through integrated petrographical, mineralogical, and geochemical analyses (encompassing major, trace, and REEs). This multi-method approach seeks to elucidate the physicochemical conditions prevailing during its deposition and reconstruct its subsequent diagenetic history.

### Sampling and analytical techniques

**Fig. 2:**
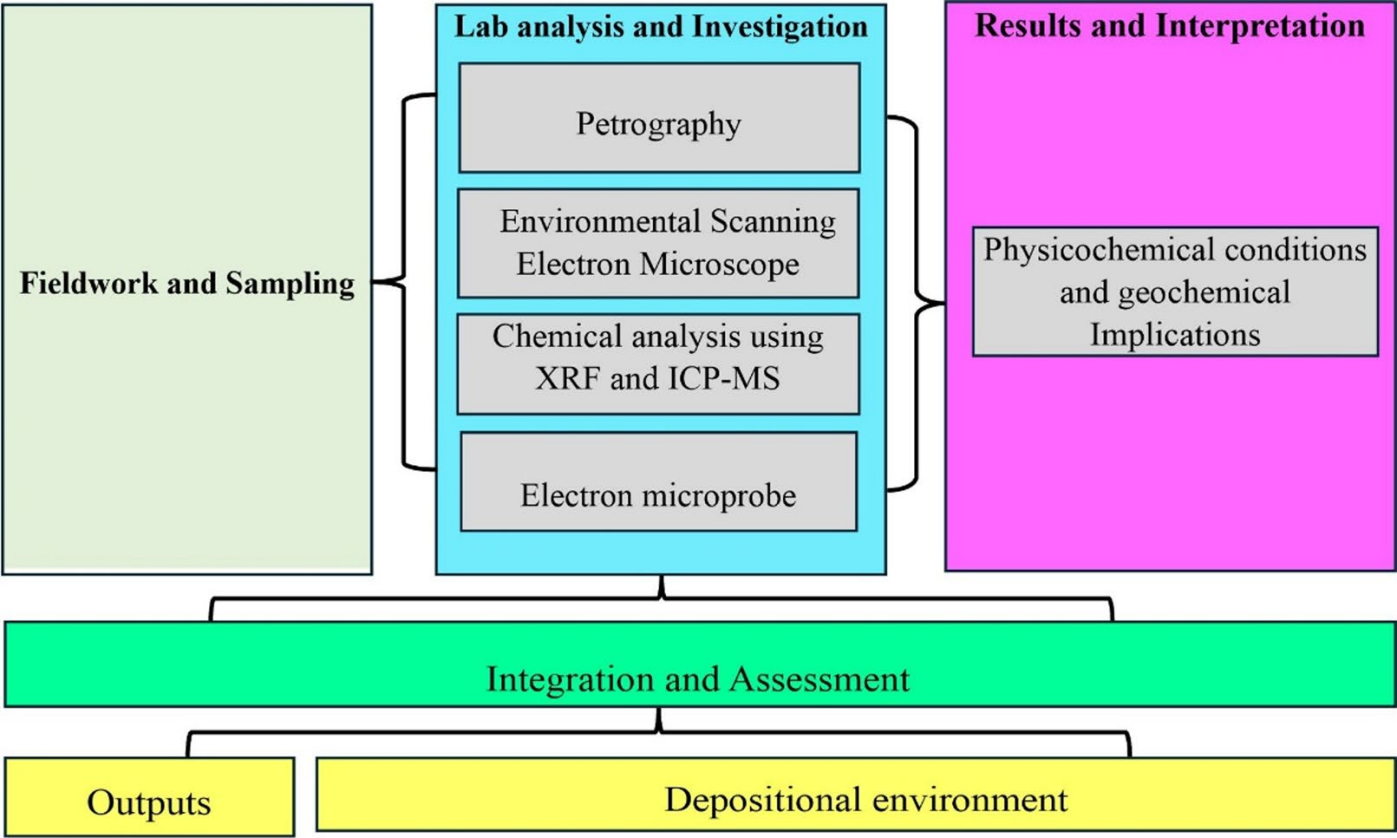
Flowchart showing methodology and analytical techniques.

A total of seventeen samples were gathered from the Lower Phosphorite Member of the Duwi Formation exposed at the studied locality. They were arranged regularly, 200 m apart, from the southern exposure to the northern one for about 3 km. Environmental Scanning Electron Microscope (ESEM, Phillips XL-30) utilized to detect the predominant elements of phosphorite at Nuclear Materials Authority laboratories. Eleven samples were prepared for petrographic (polarizing microscope to determine their mineral constituents) and chemical (major, trace, and REEs) analysis. Samples underwent XRF and ICP-MS analysis at the Geo Analytical Lab (USA). A Thermo ARL XRF Spectrometer analyzed major and key trace elements, using the USGS standard GSP2 (650 C) for calibration (Supplementary material). Analytical precision and accuracy, which were controlled through international reference materials and replicates, are 1 wt% for majors and 2–5 wt% for traces and REEs. Loss on ignition (LOI) was derived from mass loss after 1000 °C ignition. Mineral chemistry data were acquired via electron microprobe (JEOL JXA-8500 F) at 15 kV and 20 nA beam current, with calibration against natural and synthetic mineral standards (Fig. 2).

### Abu tartur geology

**Fig. 3:**
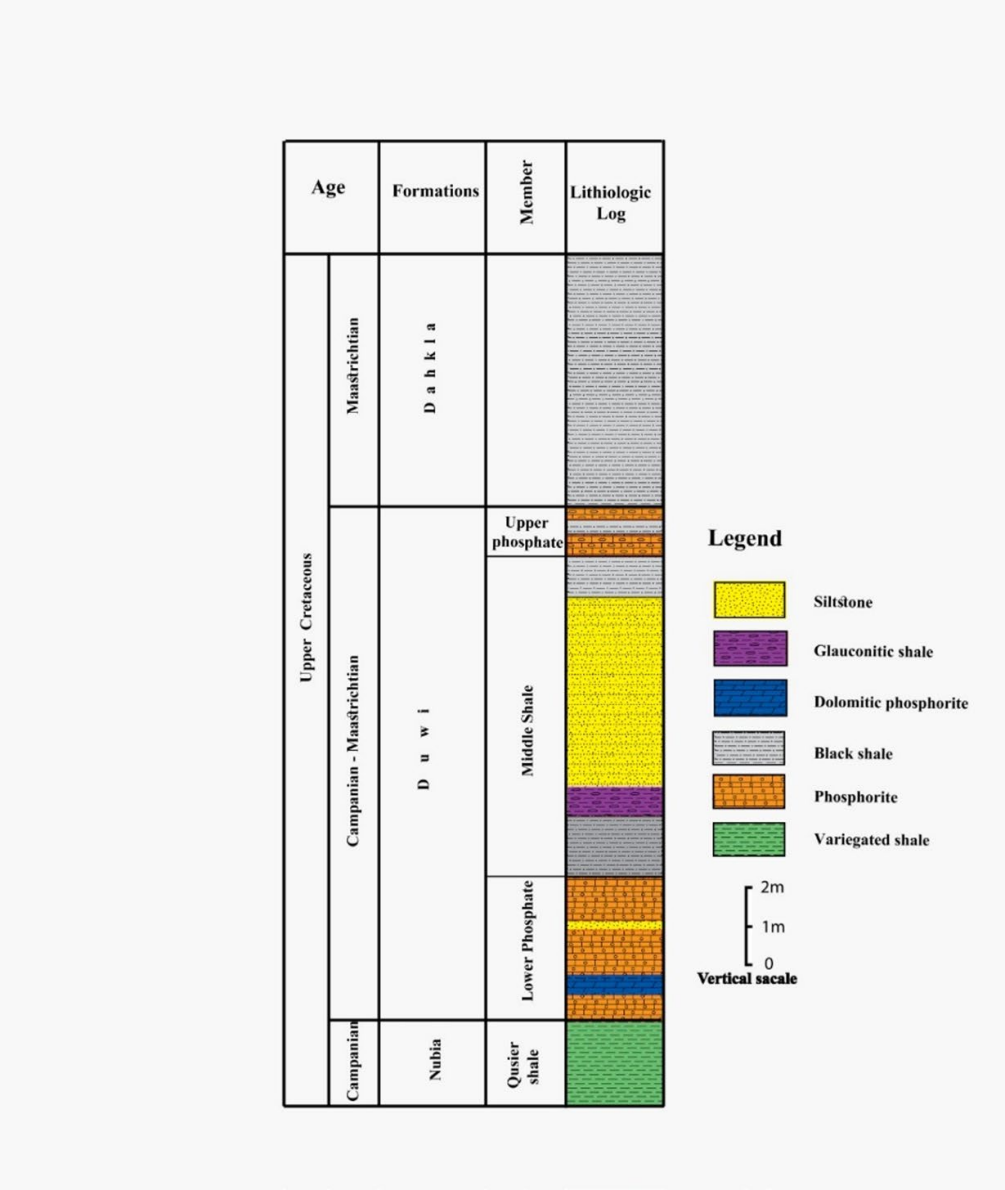
General lithostratigraphic section of the Abu Tartur area^17^.

**Fig. 4:**
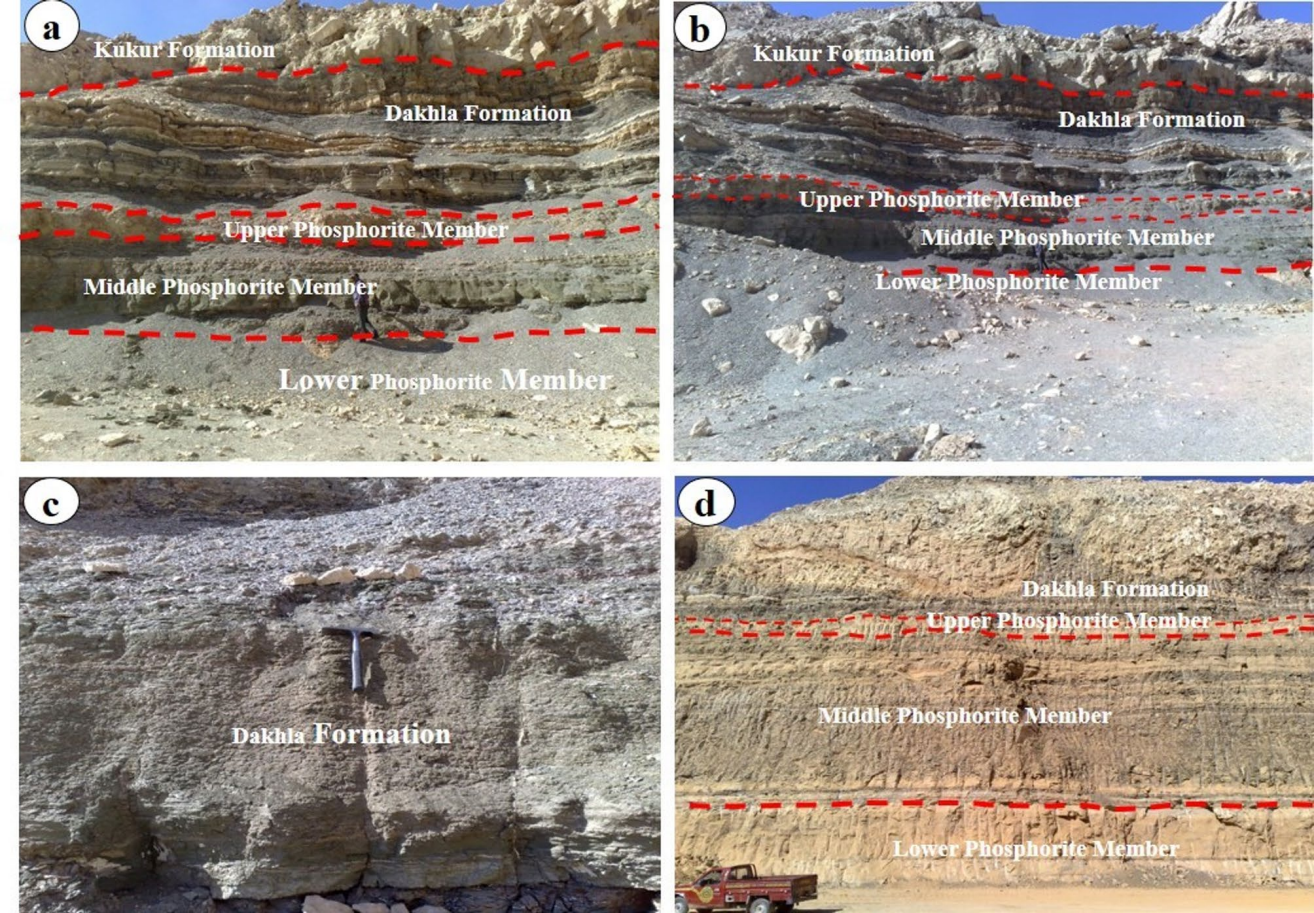
**(a**,** b**,** c**,** and d)** Photographs showing the lower phosphorite, middle shale, and upper phosphorite members of Duwi Formation at Abu Tartur area.

The Abu Tartur is extended in an east-west direction about 13 km long, Central Abu Tarur Plateau’s southern scarp boundary, ~ 15 km north of the Dakhla-Kharga road, within latitude 25°12′ − 25°41′ N and longitude 29°30′ − 30°8′ E (Fig. 1)^20^. The Duwi Formation underlies the marine shales (Dakhla Formation) that was deposited during the middle Maastrichtian and overlies the Qusseir Formation of Campanian fluvial shale. And hence, the Duwi Formation deposition marks the onset of the late Cretaceous marine transgression in Egypt^12^. In the Abu Tartur area, phosphorite beds are exposed within the Duwi Formation (Fig. 1). Following^14,18^, the Duwi Formation is subdivided into three members: Lower Phosphorite Member (avg. 2.8 m; 2–3.5 m): Moderately hard, coarse phosphatic sandstone intercalated with dolomitic phosphorite and grey shale lenses (0.25–0.8 m thick). This unit hosts the phosphorite beds (Figs. 3 and 4a-d). Middle Shale Member (avg. 7.8 m; 7–8.3 m) is consists of soft, laminated, organic-rich, black shale, and siltstone (Figs. 3 and 4a-d). Upper phosphorite Member (avg. 1 m; 1–1.2 m): is composed of coarse glauconitic sandstone, dolomitic phosphorite beds intercalated with hard black shale lenses (Figs. 3 and 4a-d).

## Results and interpretation

### Petrography

**Fig. 5:**
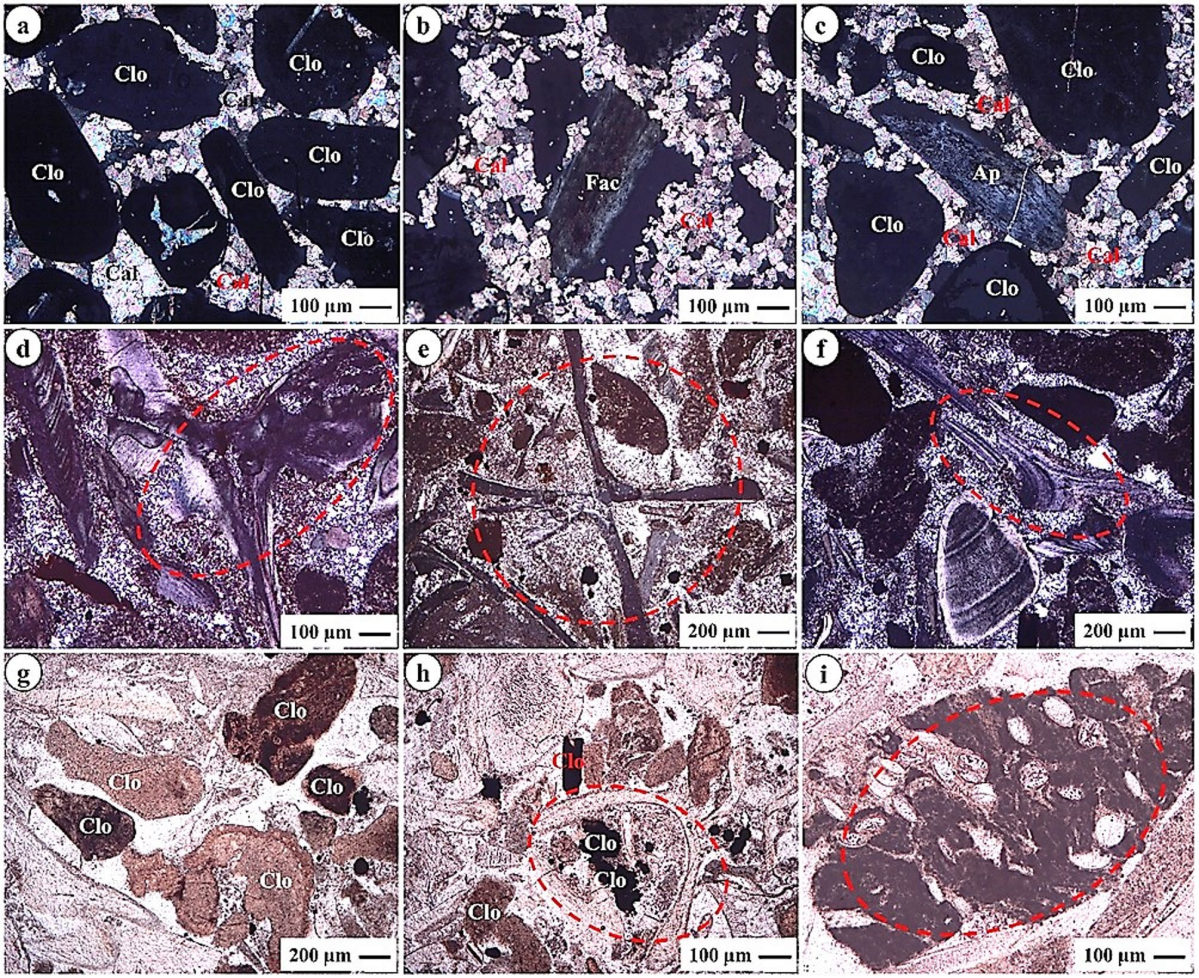
Photomicrographs show: **(a)** Collophane (Col) surrounded by calcite (Cal); **(b)** Cavity behind francolite (Frc); **(c)** Bone fragment replaced by apatite (Ap); **(d)** Bone fragment replaced partially by amorphous silica; **(e)** Bone fragment embedded in carbonate cement; **(f)** Shark teeth; **(g)** Aggregate of collophane; **(h)** Shell fragments; and **(i)** Bizolite (oval) shell fragment (iron oxide).

**Fig. 6:**
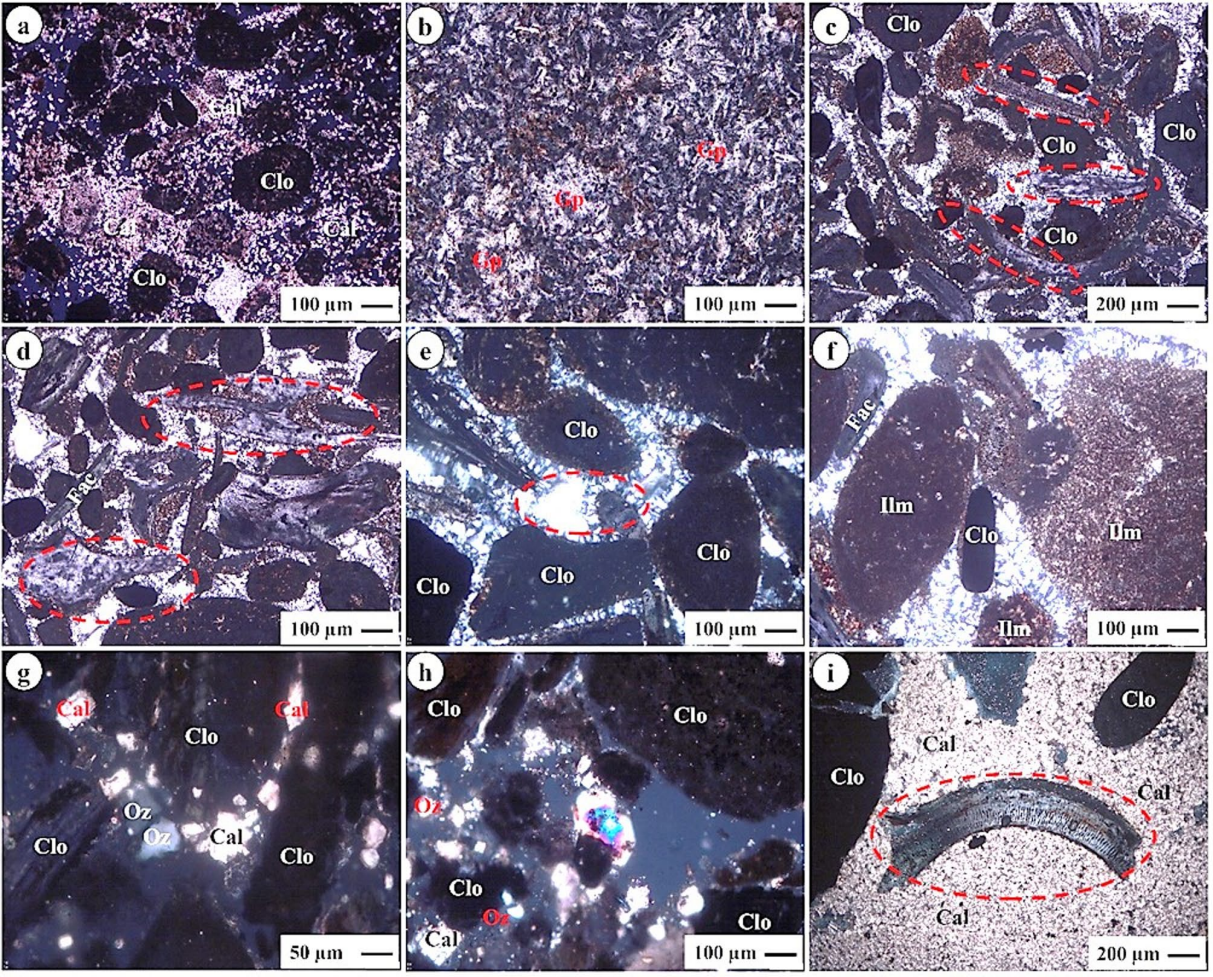
Photomicrographs show: **(a)** Collophane (Col) with microsparite (Cal); **(b)** Gypsum (Gp); **(c)** Bone fragment and collophane embedded in silica groundmass; **(d)** Bone fragment; **(e)** Comb structure of amorphous silica; **(f)** Ilmenite (Ilm) iron oxide; **(g)** Clastics from quartz (Qz) and carbonate; **(h)** Clastics from quartz (Qz) and carbonate; **(i)** Bone fragment and collophane embedded in microsparite cement.

The phosphorite samples investigated from the Abu Tartur area were subjected to detailed microscopic analysis, revealing two principal constituents: phosphatic and non-phosphatic components. The former components primarily include apatite, fish bone fragments, and shark teeth, as illustrated in Figs. 5c- f, amp and h and 6c, d, amp and i. Apatite is the primary phosphate mineral and serves as a crucial indicator of phosphorus concentration in the samples. Fish bone fragments and shark teeth further signify the biological contributions to the sediment, reflecting a diverse marine ecosystem. Additionally, fish bone fragments are characterized by their elongated to sub-angular shapes, showcasing their variability in size and shape, which underscores the clastic allochemical nature of the sediment. The phosphate grains are predominantly angular to sub-rounded and elongated in shape, exhibiting a yellowish-brown color, as shown in Figs. 5a- h and 6a- d, amp and i. This coloration may result from the oxidation of pyrite, (sometimes producing bizolite; altered apatite) a common mineral in sedimentary environments, leading to the formation of iron oxyhydroxides, as noted by^18^. The angularity of the phosphate grains indicates limited transport, suggesting they were likely deposited close to their source. The presence of these components suggests that the depositional environment was rich in marine life, providing essential nutrients that led to the accumulation of phosphates.

In contrast, the non-phosphatic components consist of dolomite, calcite, gypsum, and iron oxides, which function as cementing materials within the phosphorite matrix, as depicted in Figs. 5a, amp and i and 6a, & f-h. Dolomite and calcite are carbonate minerals be fundamentally important in binding the phosphatic grains, enhancing the structural integrity of the sedimentary rock. Gypsum, indicative of evaporative conditions, suggests fluctuations in water levels during the sedimentation process. Iron oxides, formed through the oxidation of iron-bearing minerals, contribute to the overall coloration and geochemical properties of the rock.

### Mineralogy

**Fig. 7:**
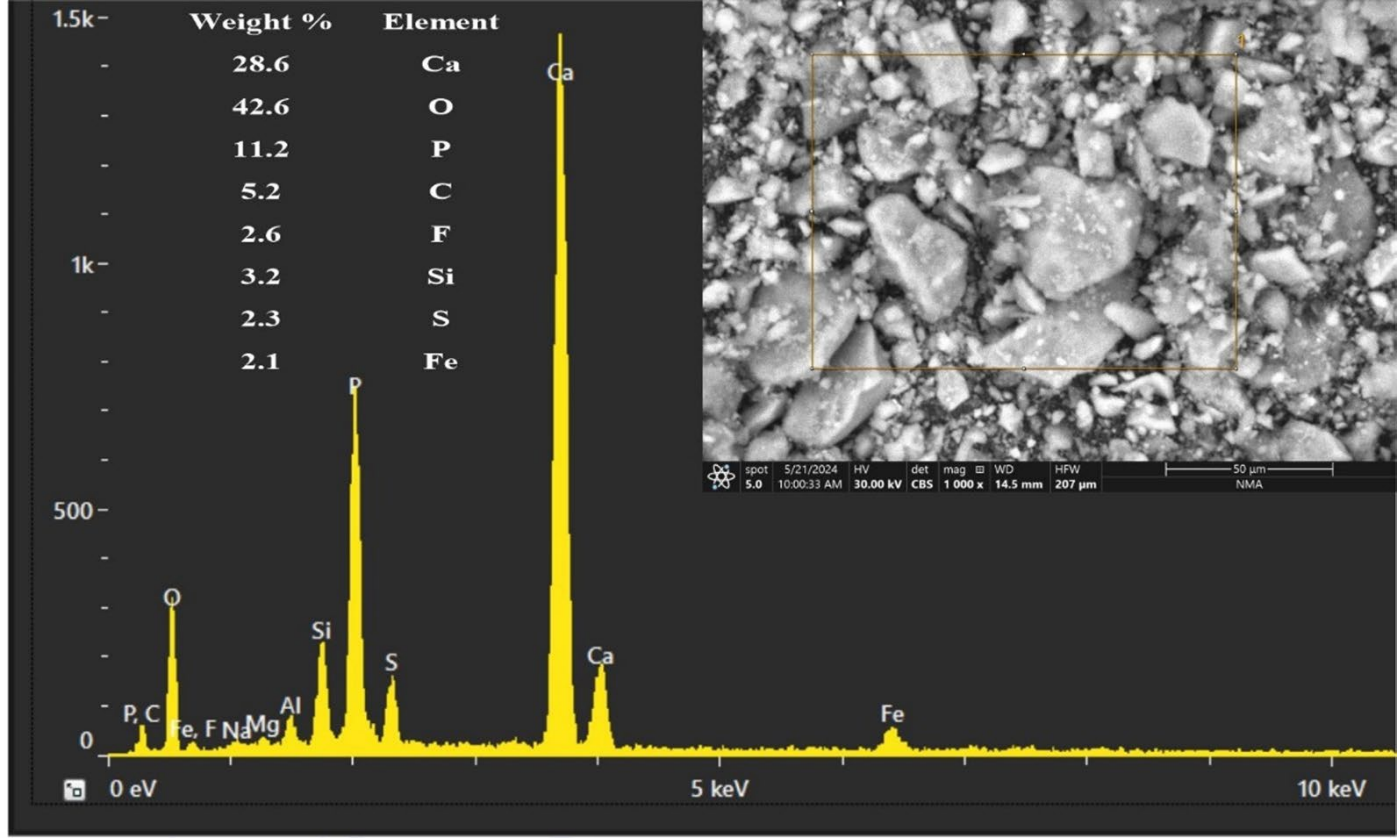
ESEM and EDX analysis of the phosphorite.

Utilizing the Environmental (ESEM, Phillips XL-30) Scanning Electron Microscope of phosphorite. The ESEM results show that the investigated phosphorite has Ca = 28.6%, *P*= 11.2%, F = 2.6%, and Fe = 2.1% (Fig. 7). The high concentrations of Ca, P, and F are characteristic of fluorapatite, confirming it as the dominant phosphate mineral. The significant Fe content suggests the presence of associated iron oxides or sulfides, which is common in phosphorites and can influence their chemical behavior. This is aligns with the petrographic section (Figs. 5 and 6).

### Apatite chemistry

Apatite grains from the examined phosphorite were analyzed and given in Supplementary table S1. Apatite is essentially a Ca-phosphate, which is present in the different rocks of the earth’s crust with a structure that can accommodate elements such as Na, Fe, Mn, Y, and REEs, as well as halogens like F and Cl^21,22^. Apatite grains have significant chemical variation in their composition. They have high contents of P_2_O_5_ (20.97–35.28 wt%; avg. 31 wt%) and CaO (31.99–55.79 wt%; avg. 48.81 wt%). Wide variations also were observed in their FeO* (0.13–7.69 wt%; avg. 1.65 wt%) and F (1.29–7.32; avg. 3.95 wt%).

The chemical variation in the apatite grains provides key insights into their formation history. The high average concentrations of P_2_O_5_ and CaO confirm a fluorapatite, but the wide ranges in F, and particularly FeO*, are highly diagnostic. The substantial FeO* content (up to 7.69 wt%) is a critical indicator of redox conditions. This FeO* content was likely incorporated under suboxic to anoxic conditions during early diagenesis. Under such conditions, iron is soluble and can be co-precipitated with calcium phosphate^23^.

Apatite is a major host for REEs in sedimentary environment. During diagenesis, apatite typically precipitates from fluids that are enriched in REEs released from the breakdown of organic matter and detrital minerals (e.g., monazite). The wide range in P₂O₅ (20.97–35.28 wt%) and CaO contents suggests substantial chemical substitution, a common feature of diagenetic apatites. The incorporation of REEs³⁺ into the apatite structure occurs primarily via a coupled substitution mechanism where REE³⁺ + Na⁺ replace 2Ca²⁺, which is charge-balanced by the substitution of SiO_4_⁴⁻ for PO_4_³⁻^23–25^. Furthermore, the F content (1.29–7.32 wt%) also shows considerable variation, which reflects the degree of post-depositional diagenetic alteration, likely sourced from the alteration of clay minerals. The high average F content (avg. 3.95 wt%) indicates that the diagenetic fluids were F-rich. Additionally, the variation in F likely reflects local heterogeneity in fluid composition and water-rock interaction processes during progressive diagenesis^25^.

### Phosphorite geochemistry

#### Major oxides content

The collected phosphorite samples were chemically analyzed to determine their major oxide contents (Table 1). The predominant oxides in the analyzed phosphorite deposits are CaO (16.60–44.86 wt%; avg. 35.83 wt%) and P_2_O_5_ (11.18–33.33 wt%; avg. 25.8 wt%) with high average of detrital elements like SiO_2_ (9.08 wt%), Al_2_O_3_ (1.74 wt%), and TiO_2_ (0.09 wt%) relative to seawater- derived phosphorite^1^.

In contrast, Na₂O and K₂O occur as minor constituents, averaging 0.57 wt% and 0.13 wt%, respectively. Major oxide contents of the phosphate ores can be summarized as: CaO > P_2_O_5_ > LOI > SiO_2_ > Fe_2_O_3_ > Al_2_O_3_ > MgO > Na_2_O > K_2_O > MnO > TiO_2_. Traditionally, CaO and P₂O₅ represent the principal constituents of apatite minerals, whereas Fe₂O₃ and MgO are associated with the presence of hematite and dolomite, respectively. The variation in P₂O₅ content among the samples (11.18–33.33 wt%) likely reflects diagenetic and geochemical processes that led to partial replacement of P₂O₅ by other components such as SiO₂, MgO, and Fe₂O₃.

**Table 1:** Chemical (major, trace, REEs, and some of geochemical parameters and elemental ratios) analysis of the Abu Tartur phosphorite.

S.No.	PAT10	PAT13	PAT15	PAT18	PAT20	PAT26	PAT29	PAT30	PAT33	PAT36	PAT38
	Major elements (wt%)										
SiO_2_	5.73	7.51	12.25	7.00	16.48	10.33	7.53	4.60	7.13	17.41	3.89
TiO_2_	0.04	0.09	0.14	0.08	0.24	0.12	0.06	0.05	0.05	0.06	0.04
Al_2_O_3_	1.01	1.48	3.25	1.20	5.08	2.74	0.98	0.64	0.89	1.17	0.73
Fe_2_O_3_	8.72	11.80	6.64	3.48	13.23	11.71	2.79	2.67	2.91	2.24	3.46
CaO	33.82	31.04	32.02	42.62	16.60	26.43	42.36	44.86	42.46	37.47	44.40
MgO	3.48	0.36	0.82	0.31	0.92	0.56	0.59	0.78	0.59	0.28	0.37
Na_2_O	0.42	0.48	0.50	0.70	0.26	0.41	0.67	0.72	0.68	0.69	0.72
P_2_O_5_	20.25	22.24	22.70	31.65	11.18	18.72	31.34	33.20	31.42	27.77	33.33
K_2_O	0.08	0.09	0.21	0.10	0.30	0.16	0.11	0.09	0.10	0.16	0.08
MnO	0.16	0.09	0.08	0.12	0.06	0.07	0.12	0.13	0.12	0.09	0.11
LOI	16.65	20.74	17.53	8.70	30.30	22.72	8.56	7.38	8.02	7.48	8.27
	**Trace elements (ppm)**										
Sc	14.28	19.99	17.71	24.63	18.54	19.05	26.66	25.25	26.95	19.92	26.14
V	53.83	65.41	88.78	54.24	185.97	81.50	52.70	48.90	49.40	19.00	43.02
Cr	13.43	22.02	48.09	25.66	96.89	36.15	29.44	23.85	25.41	8.98	16.68
Co	13.87	11.63	11.66	19.71	7.14	10.00	18.66	18.59	18.48	156.15	19.79
Ni	77.80	90.89	62.09	40.35	122.35	94.76	38.40	39.55	38.19	172.69	34.10
Cu	3.81	5.31	3.74	4.11	19.18	8.83	2.87	3.54	5.11	3.16	4.21
Zn	32.36	27.59	29.34	30.61	37.47	28.77	33.01	112.98	33.68	151.09	38.60
Ga	2.27	4.01	5.38	5.48	7.38	4.50	5.78	5.09	4.83	9.90	4.22
Rb	4.11	6.15	4.41	5.13	7.41	7.02	5.07	4.74	3.17	4.41	3.17
Sr	1115.5	1177.2	1332.3	1648.4	849.73	1123.6	1572.2	1571.7	1561.9	1346.4	1554.02
Y	95.13	119.94	125.90	218.49	90.10	112.87	245.75	253.91	235.92	310.20	194.27
Zr	136.62	150.69	169.85	199.76	145.94	142.13	197.82	191.62	198.94	164.73	187.14
Nb	12.08	15.20	16.18	24.85	17.54	16.99	28.50	27.78	27.63	41.12	22.25
Mo	47.01	32.06	44.96	9.86	37.27	30.83	5.75	5.31	5.99	5.56	9.57
Ba	43.86	58.44	66.27	73.08	61.16	51.14	61.26	56.24	64.99	67.79	54.48
Hf	0.51	0.66	0.73	0.51	2.10	0.66	0.76	0.55	0.75	0.57	0.34
Pb	251.67	288.81	212.69	98.71	326.21	288.50	66.89	77.10	70.99	280.68	109.21
Th	7.98	6.42	5.47	4.86	9.99	3.87	5.98	10.53	7.34	6.76	5.74
U	22.92	22.01	37.55	31.40	15.66	19.99	29.45	27.00	28.59	35.53	28.06
La	82.04	136.46	114.98	169.70	97.78	101.64	190.72	161.65	179.42	303.18	160.44
	**REEs (ppm)**										
Ce	157.21	255.77	220.49	313.32	183.59	192.30	350.01	299.03	326.17	582.65	295.04
Pr	19.82	32.18	27.95	40.35	22.67	24.09	45.26	38.32	42.44	67.93	37.39
Nd	83.12	138.47	116.04	174.65	93.89	102.98	196.15	165.01	183.39	277.83	161.24
Sm	16.67	27.06	23.73	35.87	18.68	20.72	39.87	33.33	37.07	53.55	33.19
Eu	4.40	7.01	6.14	9.63	4.23	5.61	10.86	9.36	10.13	14.61	9.02
Gd	17.49	28.87	24.80	40.11	18.15	22.24	44.78	37.66	41.71	58.52	36.40
Tb	2.62	4.06	3.45	5.77	2.91	3.25	6.35	5.42	6.01	8.23	5.23
Dy	16.24	25.88	22.02	36.61	16.55	20.40	40.04	33.68	37.92	50.69	32.86
Ho	3.27	5.53	4.40	7.65	3.53	4.36	8.43	7.10	7.95	10.16	6.92
Er	9.63	16.22	13.06	22.61	10.61	12.92	25.07	20.90	24.01	29.10	20.31
Tm	1.36	2.27	1.80	3.07	1.46	1.79	3.45	2.88	3.28	3.80	2.78
Yb	8.88	14.51	11.37	19.54	9.50	11.85	21.78	18.22	20.72	22.68	17.70
Lu	1.36	2.24	1.73	2.93	1.44	1.72	3.21	2.77	3.10	3.30	2.68
	**Geochemical ratios and parameters**										
ΣREE	424.12	696.52	591.94	881.81	485.00	525.85	985.99	835.35	923.32	1486.23	821.19
ΣLREE	358.86	589.94	503.18	733.90	416.61	441.72	822.01	697.35	768.49	1285.14	687.30
ΣHREE	65.26	106.58	88.76	147.91	68.39	84.13	163.98	138.00	154.83	201.09	133.89
ΣLREE/ΣHREE	5.50	5.54	5.67	4.96	6.09	5.25	5.01	5.05	4.96	6.39	5.13
Y/Ho	29.06	21.69	28.59	28.55	25.49	25.89	29.15	35.76	29.66	30.52	28.07
CaO/P_2_O_5_	1.67	1.40	1.41	1.35	1.48	1.41	1.35	1.35	1.35	1.35	1.33
V/Ni	0.69	0.72	1.43	1.34	1.52	0.86	1.37	1.24	1.29	0.11	1.26
V/Cr	4.01	2.97	1.85	2.11	1.92	2.25	1.79	2.05	1.94	2.12	2.58
V/V + Ni	0.41	0.42	0.59	0.57	0.60	0.46	0.58	0.55	0.56	0.10	0.56
V/V + Cr	0.80	0.75	0.65	0.68	0.66	0.69	0.64	0.67	0.66	0.68	0.72
Rb/Sr	0.00	0.01	0.00	0.00	0.01	0.01	0.00	0.00	0.00	0.00	0.00
V/Sc	3.77	3.27	5.01	2.20	10.03	4.28	1.98	1.94	1.83	0.95	1.65
Ni/Co	5.61	7.82	5.33	2.05	17.14	9.48	2.06	2.13	2.07	1.11	1.72
Th/U	0.35	0.29	0.15	0.15	0.64	0.19	0.20	0.39	0.26	0.19	0.20
U/Mo	0.49	0.69	0.84	3.18	0.42	0.65	5.12	5.08	4.77	6.38	2.93
(La/Sm)_N_	0.72	0.73	0.70	0.69	0.76	0.71	0.70	0.71	0.70	0.82	0.70
(Gd/Yb)_N_	1.19	1.20	1.32	1.24	1.16	1.14	1.24	1.25	1.22	1.56	1.25
(La/Yb)_N_	0.68	0.69	0.75	0.64	0.76	0.63	0.65	0.66	0.64	0.99	0.67
(Sm/Yb)_N_	0.95	0.95	1.06	0.93	1.00	0.89	0.93	0.93	0.91	1.20	0.95
(La/Lu)_N_	0.68	0.69	0.75	0.65	0.77	0.66	0.67	0.66	0.65	1.03	0.67
Pr/Pr*	1.01	1.00	1.02	1.01	1.01	1.00	1.01	1.01	1.01	0.99	1.00
Ce/Ce*	0.88	0.86	0.88	0.84	0.88	0.87	0.84	0.85	0.83	0.91	0.85
Eu/Eu*	1.21	1.18	1.19	1.20	1.08	1.23	1.21	1.24	1.21	1.23	1.22
(Sm/Pr)_N_	1.34	1.34	1.35	1.41	1.31	1.37	1.40	1.38	1.39	1.25	1.41
Y/Y*	1.04	0.80	1.02	1.04	0.94	0.95	1.07	1.31	1.08	1.09	1.03

The high content of this ratio may have been ascribed to the substitution of PO_4_ by CO_3_ and/or the presence of calcite/dolomite (enrichment of calcareous cement) in whole rocks^3^. The microscopic study showed that the replacement of phosphorite particles by cementing materials reduces phosphorite grade. For quality assessment, industry widely uses the weight percentages of P_2_O_5_, CaO, Fe_2_O_3_, and MgO^26^. Based on Zidan^27^ classification of phosphorites ore (high grade: >27 wt% P₂O₅; medium grade: 23–27 wt% P₂O₅; low grade: <23 wt% P₂O₅), the majority of the studied Abu Tartur phosphorite lie within high-grade.

The examined phosphorite, compared with other phosphorites, have high content of P_2_O_5_ and Fe_2_O_3_ relative to those of Hamadat^19^, El Ziat^27^, El dehous^18^, Abu Tartur^28^, El Sibaiya phosphorite^29^, Mazıdagr phosphates (Turkey)^3^, Hazm Al-Jalamis^1^. and Bled El Hadba^26^ (Table 2). The observed variation could be attributed to subtle differences in the depositional environment or geochemical processes.

**Table 2:** Correlations between average concentrations of the examined phosphorite with others.

Areas	CaO	*P*_2_O_5_	SiO_2_	Fe_2_O_3_	MgO	Al_2_O_3_	Na_2_O	K_2_O
Current study	35.83	25.8	9.08	6.33	0.82	1.74	0.57	0.13
Hamadat phosphorite^20^	41.83	25.54	9.07	1.76	0.87	0.37	0.74	0.074
El Ziat phosphorite^28^	38.72	24.75	7.3	3.38	3.4	1.7	0.92	0.2
El dehous phosphorites^19^	35.3	22.7	10.6	4.8	6.9	1.9	1.8	0.2
Abu Tartur^29^	40	25	5	3	3	2	0.6	0.14
El Sibaiya Phosphorite^30^	36	25	18	2.23	2.4	1.79	0.88	0.22
Mazıdagr Phosphates (Turkey)^4^	51.6	21.2	8.03	0.11	0.18	0.04	0.51	0.01
Bled El Hadba^27^	46.88	16.32	4.59	0.43	4.25	1.16	--	0.33
Hazm Al-Jalamis^1^	51.64	21.47	2.75	0.22	1.87	0.24	0.4	0.04

#### Trace and REEs variations

**Fig. 8:**
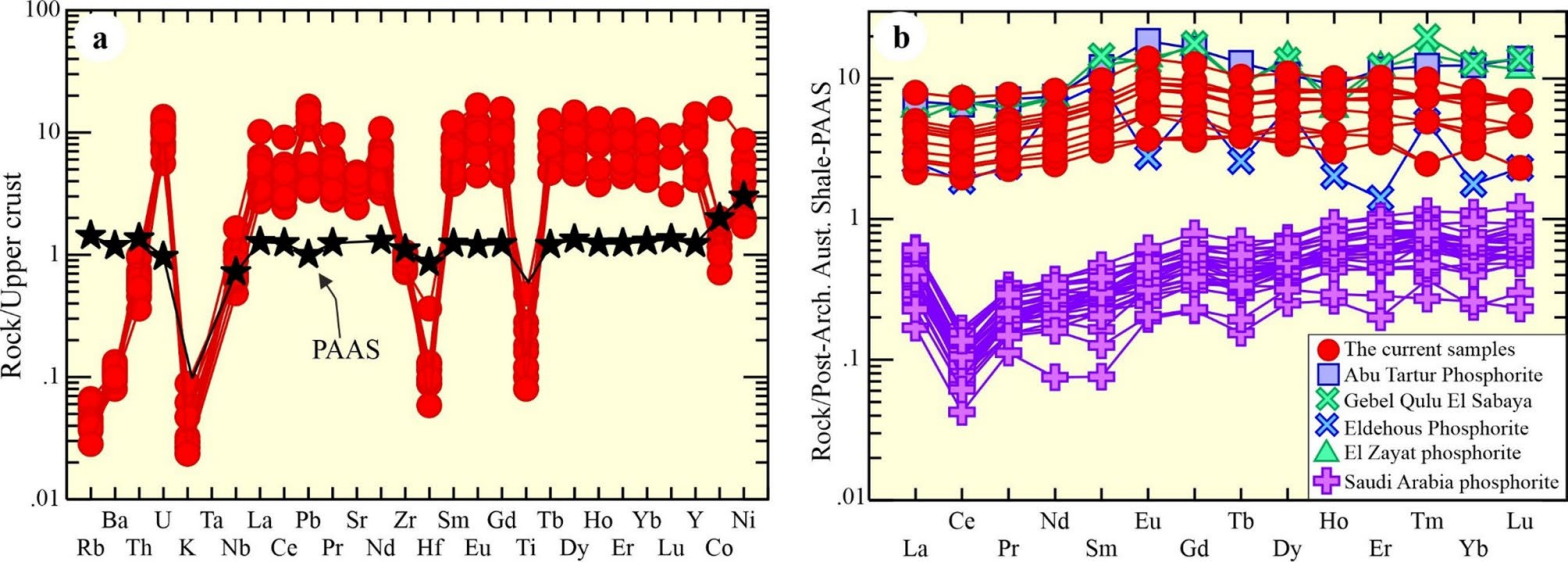
**(a)** Trace, and **(b)** REEs-normalized diagrams to UCC and PAAS, respectively^30^.

**Fig. 9:**
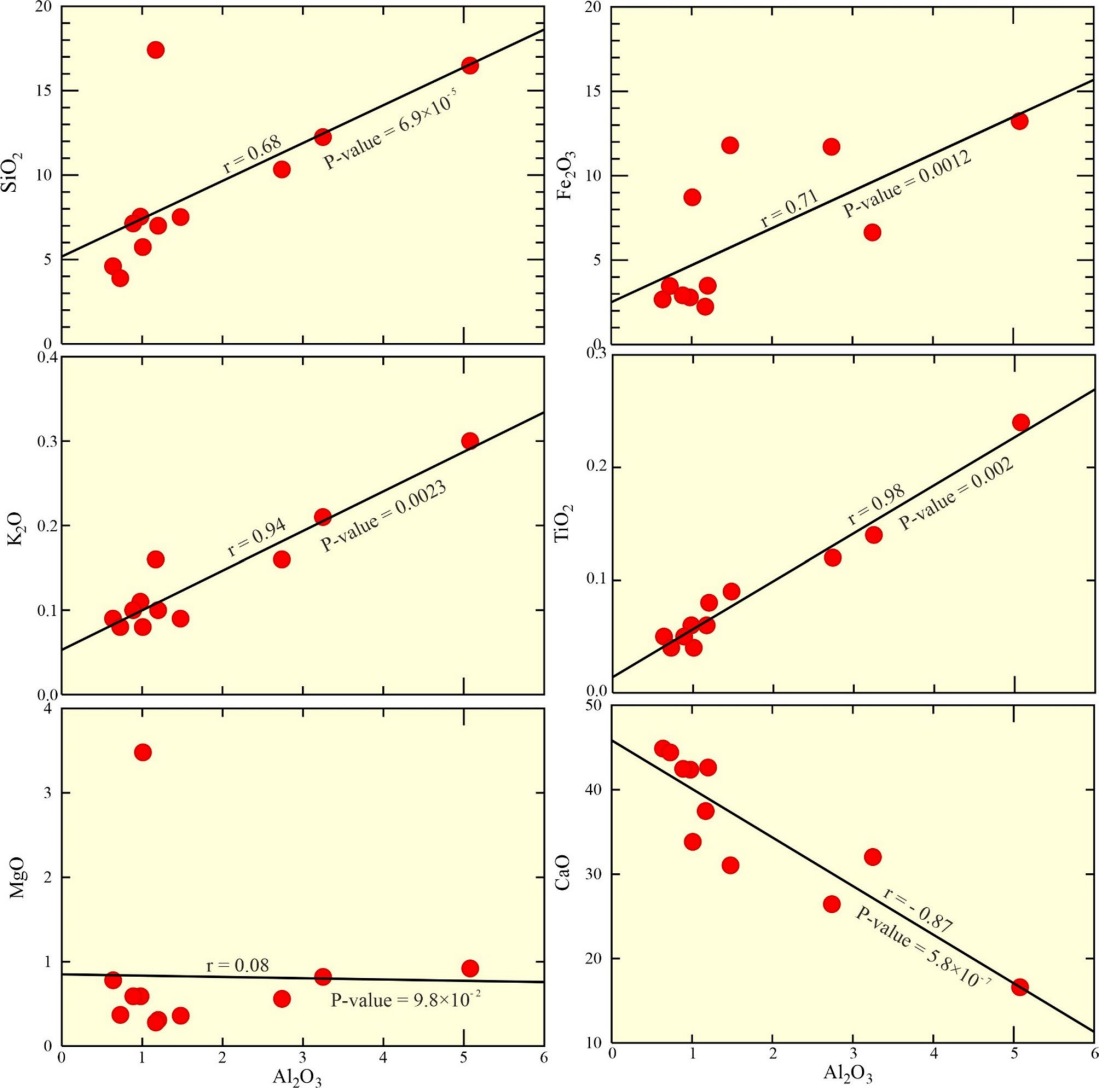
Variation diagram of Al_2_O_3_ against: SiO_2_, Fe_2_O_3_, K_2_O, TiO_2_, MgO, and CaO for the examined phosphorite deposits.

**Fig. 10:**
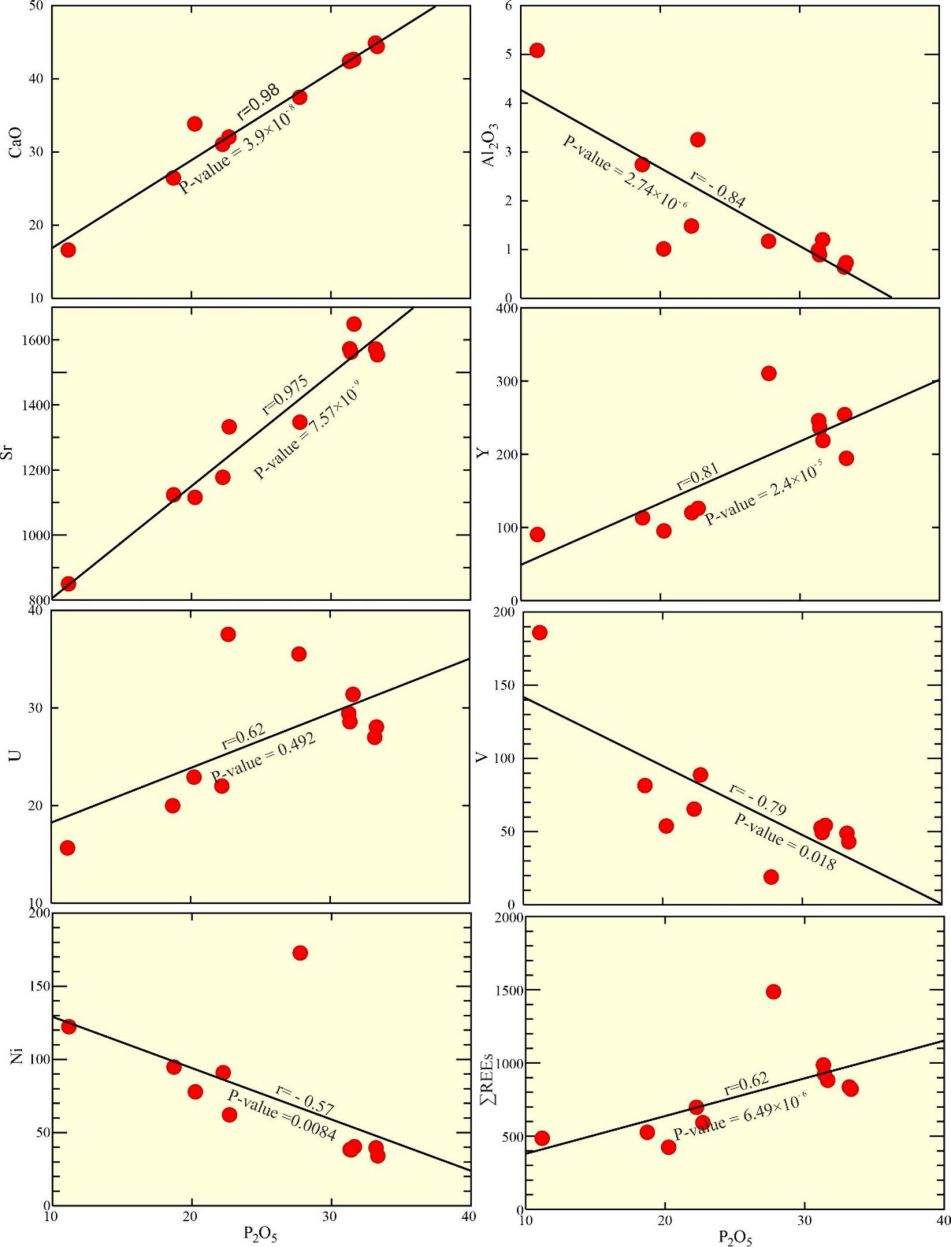
Variation diagrams of P_2_O_5_ against: CaO, Al_2_O_3_, Sr, Y, U, V, Ni, and ∑REEs for the examined phosphorite deposits.

The concentrations of trace elements in the examined phosphorite samples are given in Table 1, which shows that the Sr ranges from 849.73 to 1648.45 ppm (avg. 1350.3 ppm), Zr (136.62–199.72.62.72 ppm; avg. 171.39 ppm), V (19–186 ppm; avg. 67.5 ppm), Zn (27.6–151 ppm; avg. 50.5 ppm), Y (90.1–310.2.1.2 ppm; avg. 182 ppm), and Ba (43.86–73.86 ppm; avg. 59.88 ppm). The Sr and Ba are attributed to francolite minerals. The enrichments of detrital elements like Zr (136.62–199.72.62.72 ppm; avg. 171.39 ppm), suggesting high detrital inputs^1,6^. They represent a U-bearing phosphorite due to the enrichment of mean U content (27.11 ppm), which reveals a positive relationships with P_2_O_5_ (Figs. 8, 9, 10). They have low Th relative to U (avg. 6.8 ppm) and U/Th (avg. 4.35).

Additionally, they have a considerable concentration of compatible elements such as Cr (9–97 ppm; avg. 31.5 ppm), Ni (34–173 ppm; avg. 73.74 ppm), and Co (7.14–156 ppm; avg. 27.29 ppm). Among the large ion lithophile (e.g., Rb and Ba) elements, Sr is highly enriched compared to PAAS. While Hf, Zr, and Th are depleted compared to PAAS (Fig. 8a), U is enriched (avg. 27.11 ppm). The U concentration varies from 15.66 to 37.55 ppm, which are higher than those of the UCC (Upper Continental Crust; 2.5 ppm) and the PAAS (Post-Archean Australian Shale; 2.7 ppm)^30^, indicating that these are probably U-bearing phosphorites^17,31^. Additionally, the UCC- normalized trace elements, reveals strong negative Ti, Hf, K, and Rb anomalies (Fig. 8a).

The examined phosphorite samples exhibit remarkably high contents of ΣREEs concentration, where they range from 424.12 to 1486.23 ppm, averaging 787 ppm (Table 1). They exhibit significant enrichment of ΣLREEs (avg. 664.05 ppm) relative to ΣHREEs (avg. 122.98 ppm). The ΣLREE/HREE ratios vary between 4.96 and 6.39, which is indicated by (La/Yb)_N_ (avg. 0.7), reflecting a depletion in LREE. The slight positive Eu (Eu/Eu*) and Pr (Pr/Pr*) anomalies is calculated with mean values (1.2 and 1.01, respectively). In contrast, a slight negative anomaly of Ce (Ce/Ce*) is estimated with mean value 0.86.

PAAS-normalized REE patterns reveal clear MREEs enrichment relative to LREEs and HREEs (Fig. 8b). When the REE-normalized to PAAS^32^, the studied phosphorite is approximately comparable to that reported for the Abu Tartur deposit^17^, Eldehous phosphorite^18^, and El Sibaiya Phosphorite^29^, (Fig. 8b). Additionally, these phosphorites are inconsistent with those of Saudi Arabia phosphorite (typically seawater-derived REE)^1,6^.

## Discussion

### Implications of major oxide geochemistry

Whole-rock concentrations of SiO₂, Fe₂O₃, K₂O, TiO₂, and MgO are plotted against Al₂O₃ (Fig. 9). These correlations can be utilized to infer the source of the examined phosphorites. The SiO₂, Fe₂O₃, K₂O, and TiO₂ are positively correlated with Al₂O₃, indicating a gradual influx of these elements, likely contributing to the observed increase in glauconite and detrital clay. The correlations significance have been assessed in Supplementary Table S2.

**Fig. 11:**
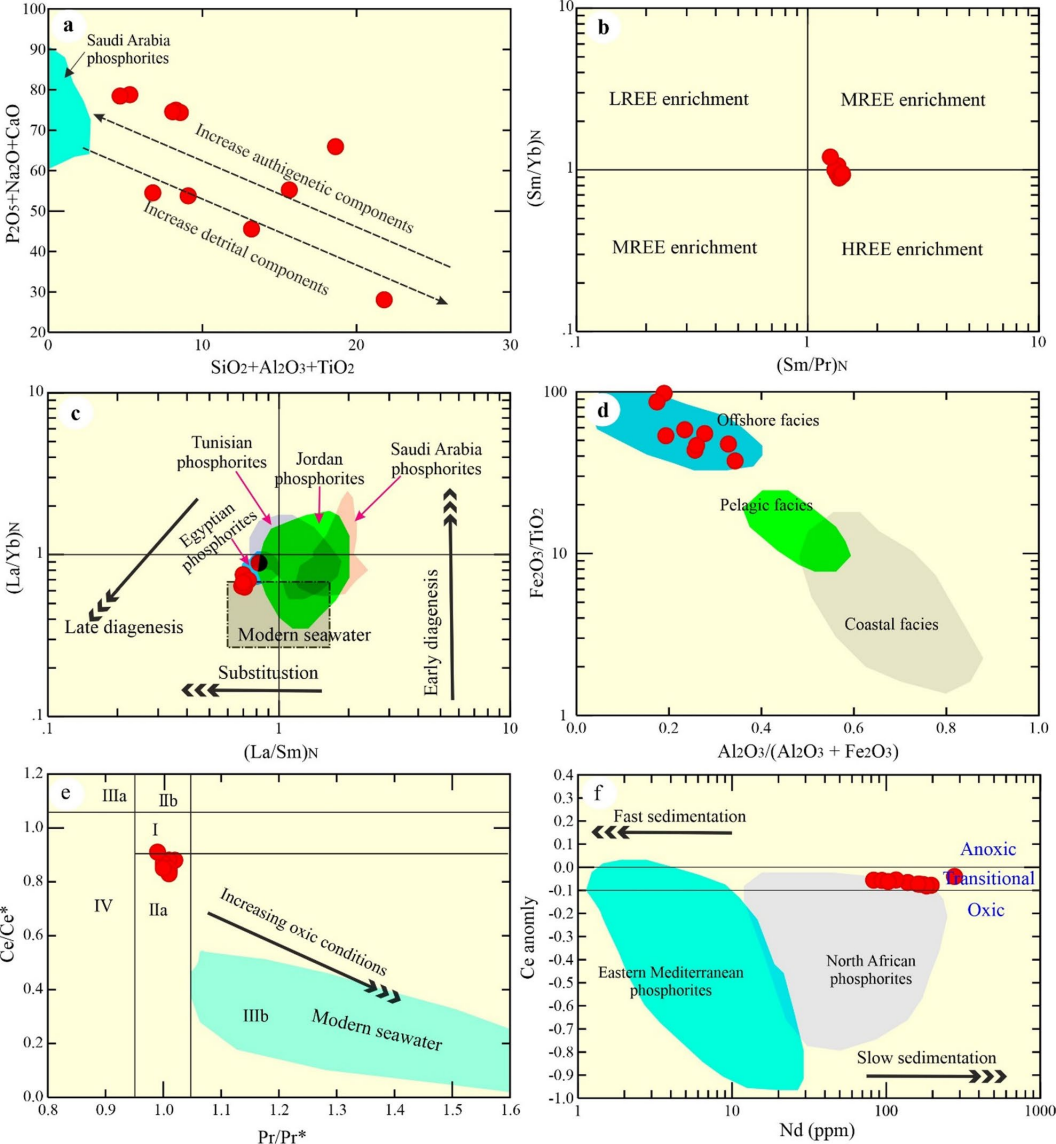
**(a)** Authigenic vs. terrestrial components relationship. Field of phosphorite from Saudi Arabia from^1^; **(b)** (Sm/Pr)_N_ vs. (Sm/Yb)_N_ REE binary diagrams that depict the evolution of depositional conditions^48^; **(c)** The (La/Yb)_N_ vs. (La/Sm)_N_ plot^56^. The studied phosphorites share a compositional similarity with Egyptian deposits^12^, but are distinct from those of seawater derived in Jordan^49^, Saudi Arabia^1^, and Tunisia^50^; **(d)** Binary provenance relationship^57^; **(e)** Pr/Pr* vs. Ce/Ce* plot^54^: Fields of I indicates no anomaly, IIa presents a positive La anomaly leading to an apparent negative Ce anomaly, IIb shows a negative La anomaly resulting in an apparent positive Ce anomaly, Field IIIa reveals a real positive Ce anomaly, IIIb indicates a real negative Ce anomaly, and IV features a positive La anomaly that obscures a positive Ce anomaly; and **(f)** Ce anomaly vs. Nd plot^55^. Fields of North African and Eastern Mediterranean phosphorites are adapted from Ahmed^1^.

The P_2_O_5_ reveals a negative relationship with the predominant of a detrital-related constituent (Al_2_O_3_; Fig. 10). High CaO content suggests a calcareous affinity of the examined phosphorite (Supplementary Table S2). In a plot of detrital versus authigenic components (Fig. 11a), the examined samples fall outside the seawater field, indicating a significant detrital input rather than a seawater-dominated origin^1,6^.

The MgO, derived from both dolomite cement and glauconite, shows a weaker relationship. In contrast, CaO is a negatively correlated with Al₂O₃, but positively correlated with P₂O₅ (Fig. 10). This, combined with P₂O₅’s negative correlation with Al₂O₃ (Fig. 10), suggests CaO is shared between calcium fluorapatite (CFA) within phosphate particles and calcareous cement (calcite and dolomite) (Figs. 5 and 6). The negative Al₂O₃-P₂O₅ & CaO relationships highlight dilution of phosphatic minerals by siliciclastic influx (Figs. 9 and 10). The correlations significance have been assessed in Supplementary Table S2.

The CaO/P_2_O_5_ ratio is used as indicator for the economic importance of these rocks (they can be more suitable for industrial applications when this ratio < 1). The examined phosphorites have CaO/P_2_O_5_ varies from 1.33 to 1.67 (> 1), minimizing their application (e.g. increase sulfuric acid consumption during H_3_PO_4_ manufacturing). This ratio reflects a considerable amount of calcite as indicated by petrographic section (as a cement material), which is supported by high LOI (avg. 14.2 wt%).

### Trace element insights into phosphorite origin and evolution

#### Sr, Y, U, REEs substitution in apatite lattice

The Sr, Y, and U trace elements are positively correlated with P_2_O_5_, as they substitute for Ca in the apatite minerals of the phosphate particles (Fig. 10). Elements like Sr, Ba, Ni, V, U, REEs, and Cr are incorporated into the crystal lattice of CFA^15^. U concentrations range from 15.66 to 37.55 ppm, averaging 27.11 ppm in the phosphate samples (Table 1). The high Sr content, may be ascribed to biologic source^1^. Moreover, the ratio of Sr/Ba is high (avg. 22.74), pointing to normal marine deposition^1^.

The high U (avg. 27.1 ppm) content relative to Th (avg. 6.81 ppm) with relatively high U/Th (4.35) value, reflecting the role of detrital input. Ca²⁺ (ionic radius 0.99 Å) is also exchangeable with Sr²⁺ (1.12 Å), Na⁺ (0.93 Å) or REEs and other analogous radius elements. In the examined phosphorites, Sr, as a trace element, is recorded at the highest concentrations, up to 1648.45 ppm, confirms marine depositional control. It is abundant in seawater oceanic with an average concentration of 8100 ppm^33^ and marine phosphorites average for 1900 ppm^26^. Furthermore^34^, established that apatite Sr composition, pointing to equilibrium deposition from fluids with different Sr contents. Therefore, the marine origin of the examined phosphorites is manifested by their elevated Sr levels. Sr displaces Ca in CFA, with Ba acting as a less common substituent^34^, indicating that the phosphorite deposits, predominantly made up of CFA^35^, have higher Sr (avg. 1350.30 ppm) and Ba (up to 73.08 ppm) concentrations.

#### V, Ni, and other elements substitution

As a result, V and phosphorus demonstrate a strong negative correlation in the phosphate samples examined (Fig. 10). This substitution can result in economically viable concentration. Likewise, Ni exhibits chemical behavior similar to that of V in the phosphorite of Abu Tartur. The relationship between Ni and P_2_O_5_is due to its small ionic radius (0.62 A°^36,37^;, Ni is less incorporated into the structure of apatite^37^. V^4+^ (0.97 A°), U^4+^ (0.98 A°), Th^3+^ (0.98 A°), and Y^3+^ (0.54 A° and 0.64 A°, respectively) can replace Fe^3+^ and Cr^3+^ (0.63 A°) or Al^3+^ (0.53 A°). Additionally, Ni^2+^ (0.69 A°) may substitute for Mg^2+^ (Fe_0−25_ Mg_0−41_) (Si_3−61_ Al_0−39_)O_10_(OH)_2_). Meanwhile, V and Th⁴⁺, Pb, and U⁴⁺ can be incorporated into the apatite structure^26,38^. Because of the similarity in valence state (1^+^) and ionic radius (1.48 A°).

### REE constraints and depositional controls

The depositional setting and formation processes of phosphorites are commonly interpreted using REEs geochemistry^1^. Phosphorites are distinguished by their exceptional enrichment in REEs compared to other marine sedimentary rocks^19,26,35,39^. Their variations in REE contents are closely associated with variations in depositional conditions, detrital inputs, pH, age and depth of water^26,40,41^. Besides seawater, porewater plays a crucial role in controlling the REE composition of authigenic carbonate fluorapatite (francolite) in marine sediments^31,41^. The main phosphate mineral in the authigenic mineral assemblage, francolite, is known for its ability to become enriched in REEs by substituting REEs for Ca ^2+^^42,43^. The elevated concentration of REEs in sedimentary phosphorites is largely due to the presence of reducing conditions within organic-bearing sediments^44^. The examined phosphorite samples have an elevated total REEs content ranging from 424.12 to 1486.23 ppm, where their concentration increases from west to east direction is probably related to depositional and diagenetic aspects.

The assessment of depositional environments^43,45,46^ and the analysis of authigenic, biogenic, and reworked phosphorite formation^47^ commonly utilize PAAS-normalized REE patterns. The PAAS-normalized REEs plot shows that the examined phosphorites characterized by slight negative Ce and positive Eu anomalies (Fig. 8b). Also, the pattern shows that these phosphorites are relatively enriched in MREEs over LREEs and HREEs as manifested by the (Sm/Pr)_N_ vs. (Sm/Yb)_N_ diagram (Fig. 11b)^44,48^. This pattern is inconsistent with typical seawater-derived phosphorites, reflecting reworked phosphorites^6^. This non-conventional distribution is not comparable to that of the sea water and many other marine phosphorites, which are characterized by a large negative Ce anomaly^41–43^ (Fig. 8b). Specifically, their patterns and anomalies, point to the influence of post-depositional processes on the REE distribution^6,12^.

The (La/Yb)_N_ ratios^41^ of phosphorite deposits (0.633–0.987; avg. 0.7 Fig. 11c) and (La/Sm)_N_ (avg. 0.72) samples, slight enrichment in the (La/Yb)_N_ ratio are observed in the examined phosphorites relative to modern seawater (0.2–0.5). The studied phosphorites share a compositional similarity with Egyptian deposits^12^, but are distinct from those of seawater derived in Jordan^49^, Saudi Arabia^1^ ((Fig. 8b), and Tunisia^50^.

The REE distribution patterns (PAAS-normalized) of modern seawater are characterized by an LREE to HREE enrichment, a distinct negative Ce anomaly, and Y/Ho values > 52 (^1,22^^49,50^,–^42^. The examined samples display none of these features, which are inconsistent with seawater origin. Sedimentological observations from the Kef Essenoun (Algeria) deposits indicate a sub-oxic to sub-reduced basin setting^51^. Based on this, recent studies^6,26,48,52^ attribute REE patterns like the weak middle REE-enriched type to the transport and redeposition of phosphorites^6,53^.

From the above, the chemical features of the examined phosphorite reveal that they were likely formed by sea-level changes during the Campanian - Maastrichtian periods from the pre-existing authigenic phosphatic in offshore (Fig. 11d) North Africa^12^.

### Redox and paleosalinity conditions from the different proxies

Redox conditions are typically assessed by analyzing REE anomalies, including Ce, Y, and Eu, which can be identified in PAAS-normalized patterns, along with certain sensitive trace elements (e.g., V, Cr, Ni, and Co). The behavior of these elements is crucial for understanding the redox environment during phosphorite precipitation^26,52^.

Among REEs, Ce is particularly valuable for assessing the redox conditions of various sedimentary environments^3^. Under oxic conditions, Ce commonly occurs in its tetravalent form (Ce⁴⁺)^43^. The uptake of Ce⁴⁺ by manganese oxides and hydroxides depletes Ce in seawater, resulting in a negative Ce anomaly^54^. Therefore, the Ce anomaly is an effective indicator of seawater redox conditions^3,43^. However, Ce anomaly values can be influenced by additional factors like elevated La concentrations. A Pr/Pr* vs. Ce/Ce* plot^54^ is applied to evaluate any possible over-estimation of Ce anomalies. Most of Abu Tartur samples are located in field IIa (Fig. 11e)^54^, providing a negative Ce anomaly, which is used as an indicator for redox conditions^48^. By using the binary plot of Nd versus Ce anomaly^55^ (Fig. 11f) indicates that the Abu Tartur phosphorites were deposited under transitional conditions and further suggests a relatively slow sedimentation rate, as reflected by the enrichment of Nd and other REEs^55^.

The depositional environment of the phosphorites can be interpreted using the (La/Sm)_N_ ratio. Low values (< 0.35) are diagnostic of apatite precipitation from suboxic to anoxic pore waters, in contrast to higher values (> 1), which point to a more oxygenated marine setting^58^. The measured mean (La/Sm)_N_ value of 0.72 in these samples therefore indicates a mixing of oxic and anoxic marine environment during formation.

The redox-sensitive behavior of europium (Eu), which is reduced from Eu³⁺ to Eu²⁺ under low-oxygen conditions, makes it a useful proxy for depositional environments^53,54,59^. The measured positive Eu anomaly in the phosphorite samples (average Eu/Eu* = 1.2) is interpreted as evidence for their formation under sub-oxic to sub-reduced conditions^6,53^.

**Fig. 12:**
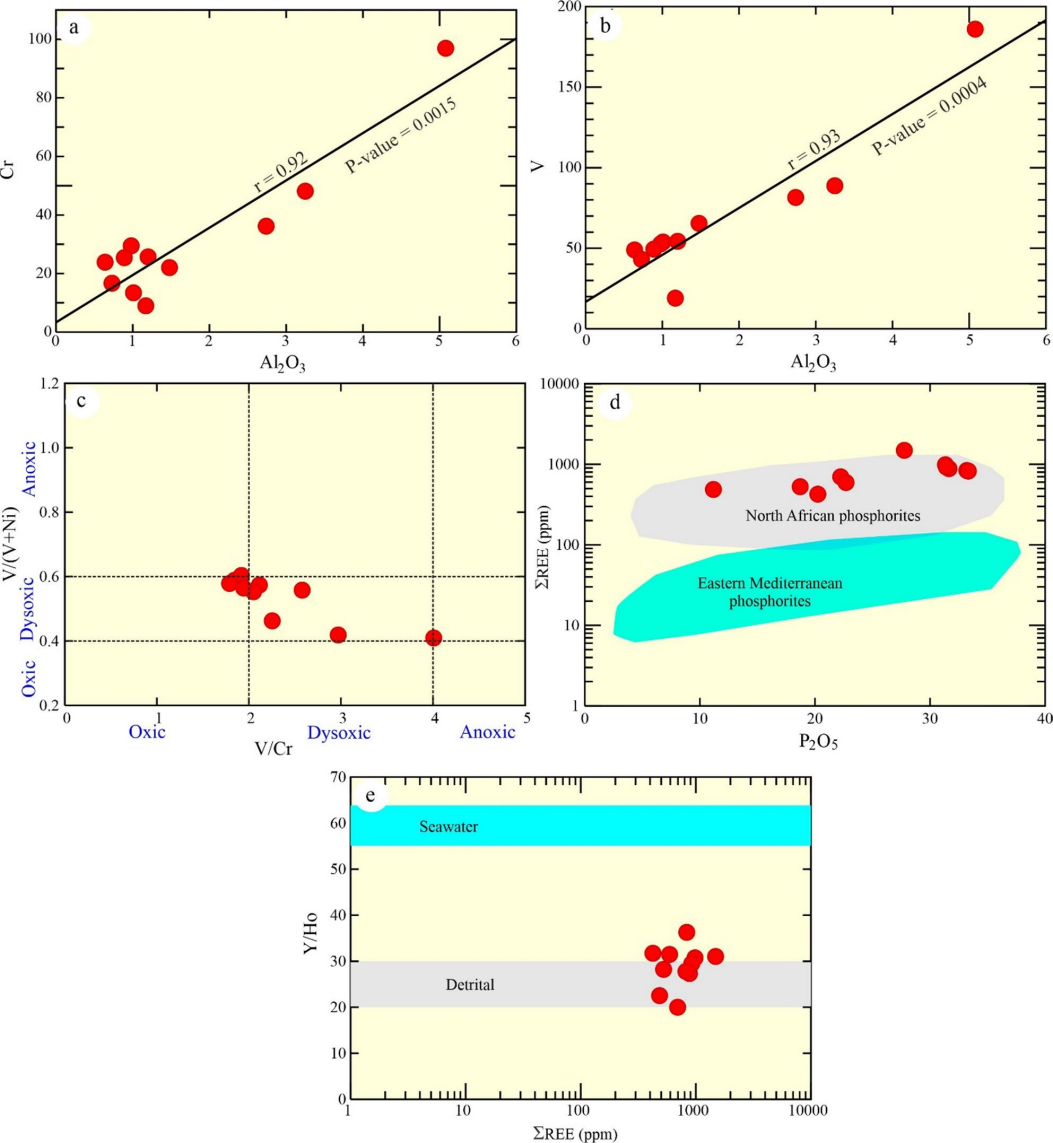
**(a-b)** Al_2_O_3_-Cr; and Al_2_O_3_-V diagram; **(c)** V/Cr vs. V/(V + Ni) plot^3,23^; **(d)** P_2_O_5_- ΣREE diagram. Fields of North African and Eastern Mediterranean phosphorites are adapted from Ahmed^1^; and **(e)** Y/Ho- ΣREE diagram^54^.

Some trace elements and their ratio can be used as an indicator for redox-sensitive substitutes such as Ni/Co, V/(V + Ni), and V/Cr, where they can reveal the global redox (paleo-ocean redox regime) conditions^3,23^. According to Table 1, the Abu Tartur phosphorites analyzed show elevated levels of V and Cr, with concentrations ranging from 19 to 186 ppm and 9 to 97 ppm, respectively. In these phosphate deposits, Cr replaces Al, which is indicated by a strong positive correlation with Al_2_O_3_ (Fig. 12a, Supplementary table S2). In contrast, V may attach to organic matter, resulting in a positive correlation with Al_2_O_3_ (Fig. 12b). According to^60^, the examined phosphates have been developed in dysoxic depositional environment, where they have 1.11–17.14 of Ni/Co (avg. 5.14), V/Cr (1.79–4.0; avg. 2.33), and V/(V + Ni) (avg. 0.49). Utilizing the V/(V + Ni) vs. V/Cr plot, the examined phosphorite straddle the fields oxic and dysoxic (Fig. 12c)^51^.

Geochemical proxies, specifically the Sr/Ba ratio, are used to infer paleo-salinity conditions. A high Sr/Ba ratio is indicative of high salinity, warm/arid climate. In this study, the phosphorite exhibit a high average Sr/Ba ratio of 22.74, which implies deposition in a saline marine and arid climate. This finding is in agreement with the recent work^15^.

The Rb/K_2_O ratio is an established proxy for paleo-salinity, with values > 6, 4–6, and < 4 indicating saline, brackish, and freshwater environments, respectively. The phosphorites in this study have a high average Rb/K_2_O ratio of 41.66, confirming deposition in a saline environment^61^. The Th/U ratio is a reliable proxy for distinguishing depositional environments, with values < 2 indicating a marine saline setting. The low average Th/U ratio of 0.27 in the studied phosphorites confirms their deposition in a saline environment^61^.

### Comparison with other phosphorites

Certain periods in geological history, particularly several intervals during the Phanerozoic age, were significantly more favorable for the formation of large phosphorite deposits. The formation of large phosphorite deposits was concentrated in specific, favorable periods during the Phanerozoic age, rather than being evenly distributed through time^6,15,23,51,58^.

The formation of large phosphorite deposits in North African (e.g., Egypt) and Eastern Mediterranean (e.g., Saudi Arabia) countries occurred during discrete intervals of the Phanerozoic. Geochemical signatures differentiate these deposits. Those from North Africa, as the examined phosphorite, are characterized by a CaO/P₂O₅ ratio of < 2, alongside excess SiO₂ and Al₂O₃, indicators of a substantial detrital input during formation. Conversely, phosphorites from Saudi Arabia exhibit a CaO/P₂O₅ ratio > 2, supporting seawater origin^1,2^.

Geochemical analysis reveals a moderate U/Th ratio (avg. 4.35) in the examined phosphorites, suggesting a depositional environment with significant terrestrial input^1,6,12^. This finding aligns with data from Tunisian phosphorites (U/Th = 5). Conversely, Eastern Mediterranean phosphorites are characterized by a higher U/Th ratio, primarily resulting from their very low thorium (Th) content.

The examined phosphorites have a high ΣREEs + Y content (avg. 969 ppm; Table 1), significantly exceeding that of Eastern Mediterranean phosphorites (Fig. 12d).

Seawater exhibits high Y/Ho ratios because Ho is scavenged from the water column more rapidly than Y. This property makes the Y/Ho ratio an effective tracer for distinguishing terrestrial from oceanic REE sources (Fig. 12e)^1,6^. Detrital-derived REEs are characterized by a lower Y/Ho ratio (25–30), while seawater-derived REEs have a higher ratio (~ 60). On a Y/Ho vs. ΣREE plot^54^, the studied phosphorites cluster with the detrital field, contrasting with Eastern Mediterranean phosphorites, which align with a seawater-derived REE source^1,6^.

Geochemical data reveal distinct depositional conditions for the phosphorite deposits. The high Nd content and seawater-range Ce anomaly in the North African phosphorites are consistent with a slow sedimentation rate, analogous to the global average^1,6,58^. Conversely, the lower Nd content in the Eastern Mediterranean phosphorites points to oxic conditions and a faster sedimentation rate. The specific samples examined in this study, characterized by high Nd, suggest an environment with mixed oxic-anoxic conditions and slow sedimentation (Fig. 11d).

### Economic potential of U, Th, and REEs

While sedimentary phosphorites are known to host U, Th, and REEs, a high Th content is uncommon as it is typically low in seawater-derived deposits. The analyzed samples are characterized by moderate U (avg. 27 ppm), significantly high Th (avg. 6.81 ppm), and high ΣREEs (avg. 787 ppm). Together, these features indicate a significant contribution from continental detrital material.

The strong positive correlation between P₂O₅ and U (Fig. 10) is attributed to U substituting for Ca in the apatite lattice and its association with carbonaceous matter. The U/P₂O₅ ratio in phosphorites is typically > 1. However, higher ratios can occur due to low U concentrations, which may reflect the specific environmental conditions during initial phosphate deposition^31^. The established infrastructure for wet-chemical phosphate processing, specifically phosphoric acid plants for fertilizer production, provides a readily available pathway for the co-recovery of U.

A major advantage of phosphorite deposits is their potential as a new resource for REEs. Specifically, REEs can be extracted as a byproduct from the conventional wet-process phosphoric acid used in the fertilizer industry^1,62^.

Financially, the established infrastructure for phosphoric acid and fertilizer production can offset the cost of REE recovery. Technically, the extraction is efficient because REEs, which occur as substitutes for Ca or P in the apatite structure, are easily liberated into solution by the mineral acids employed in the standard production process.

It is well-established that the HREEs are rarer and more expensive than LREEs. This leads to the conventional view that ore quality increases with HREE concentration, a metric typically quantified by the LREE/HREE ratio. Despite this useful metric, a comprehensive economic evaluation of REE ores remains challenging. This is partly due to the vast diversity in their geological origins and mineralogical compositions, which result in widely varying processing requirements^63^.

The examined phosphorites have a high ΣREEs + Y content (avg. 969 ppm; Table 1), significantly exceeding that of Eastern Mediterranean phosphorites (Fig. 12c), with high LREEs (avg. 646 ppm) and LREE/HREE ratio (avg. 5.42). Moreover, they have high contents of critical REEs + Y such as Nd (avg. 153.89 ppm) and Y (avg. 182 ppm) and uncritical REEs such as La (avg. 154.36 ppm).

## Conclusions

The investigation of the Lower phosphorite deposits in the Abu Tartur area, part of the Duwi Formation in Egypt, yields important insights into their geological and economic significance. The Duwi Formation is a vital component of the Mediterranean phosphorite belt, which stretches from Africa to Asia. Geochemically, the phosphorite samples exhibit a high ΣREEs concentration, with PAAS-normalized patterns showing a relative enrichment in middle REEs (MREEs) over both light and heavy REEs. The Nd and (La/Sm)_N_ values, combined with a slight negative Ce anomaly, indicate deposition in oxic and dysoxic, saline marine environment with a slow sedimentation rate. Multiple geochemical proxies consistently point to a significant detrital input. These include: low CaO/P₂O₅ ratio (< 2), coupled with elevated SiO₂ and Al₂O₃, high Zr content (136.62–199.72 ppm), low U/Th ratio (avg. 4.35 ppm), low Y/Ho ratio (avg. 28.4), and high ΣREEs + Y content (avg. 969 ppm). Collectively, these petrographic and chemical features indicate that the examined phosphorites were likely reworked from pre-existing authigenic deposits in offshore North Africa by sea-level changes during the Campanian-Maastrichtian periods.

## Supplementary Information

Below is the link to the electronic supplementary material.

Supplementary Material 1

Supplementary Material 2

## Data Availability

This article encompasses all the data analyzed.
