# Supplementary material for: Mineral and geochemical variability of the phosphorite deposits in the Duwi Formation, Western Desert, Egypt: Insights into paleoenvironment and physicochemical conditions
Source: Sci Rep. 2026 Apr 30;16:13910. doi: 10.1038/s41598-026-46266-7 (PMC13133165; doi:10.1038/s41598-026-46266-7)
Supplement: Supplementary file 1 — Supplementary Material 1 [file 41598_2026_46266_MOESM1_ESM.docx]

**Analytical Conditions**

XRF and ICP-MS analyses are carried out at the GeoAnalytical Lab, Washington State University (WSU), USA. The concentration of the major and some trace elements was determined via X-ray fluorescence (ThermoARL XRF Spectrometer). Each powdered sample was weighed, mixed with two parts di-lithium tetraborate flux, fused at 1000°C in a muffle furnace, and cooled. The resulting bead was reground, re-fused, and polished on diamond lap to produce a smooth, flat surface for analysis. The calibration standard was the reference material 650CC from the USGS standard rock powder GSP2. The detection limit for the major oxides and trace elements is available online from the GeoAnalytical Lab (<https://www.depts.ttu.edu/geosciences/geo/facilities/geoanalytical.php>). The loss on ignition (LOI) was determined by the weight difference after ignition at 1000 °C. Concentrations of REE and some trace elements were determined via ICP-mass (Agilent7700ICP-MS) spectrometry. About 50 mg power of each sample are dissolved in acid-washed Teflon containers by refluxing in hot (250 °C) 3:1 nitric and hydrofluoric acid for at least 8 hours. A working curve for instrument sensitivity was developed using a blank fused bead from the same batch of flux as used to prepare the unknowns along with USGS standards AGV-2 and RGM-2. To control for quality, additional USGS standards (DTS-2, BCR-1, G-2) were included as unknowns.

The lab's JEOL JXA-8500F electron microprobe offers high-resolution, quantitative analysis of major and minor elements at the micrometer scale. Typical detection limits for standard EPMA analysis are in the range of 30–100 parts per million (ppm), depending on the element and instrument settings. Under specialized conditions, such as using high beam currents and extended counting times on stable minerals, detection limits for trace elements can be lowered to the 5–30 ppm range.
